# Trends in quality of primary care in the United States, 2007–2016

**DOI:** 10.1038/s41598-022-06077-y

**Published:** 2022-02-07

**Authors:** Anshul Saxena, Venkataraghavan Ramamoorthy, Muni Rubens, Peter McGranaghan, Emir Veledar, Khurram Nasir

**Affiliations:** 1 Baptist Health South Florida, Miami, FL USA; 2 Florida International University, Miami, FL USA; 3 Miami Cancer Institute, Baptist Health South Florida, Miami, FL USA; 4 Department of Internal Medicine and Cardiology, Charité – Universitätsmedizin Berlin, Corporate Member of Freie Universität Berlin and Humboldt Universität zu Berlin, Augustenburger Platz 1, 13353 Berlin, Germany; 5 Division of Cardiovascular Prevention and Wellness, Department of Cardiology, Houston Methodist DeBakey Heart & Vascular Center, Smith Tower, 6550 Fannin St., Ste. 1901, Houston, TX 77030 USA; 6 Center for Outcomes Research, Houston Methodist, Houston, TX USA; 7 Baptist Health South Florida, 1575 San Ignacio Av, Suite 500, Coral Gables, FL 33146 USA

**Keywords:** Public health, Epidemiology, Outcomes research

## Abstract

During the past decade, many reforms were proposed and implemented for improving primary care in the US. This study assessed improvements in quality of primary care, using a nationally representative database. We conducted a retrospective trend analysis of National Inpatient Sample data (2007–2016). The quality of primary care was assessed using Prevention Quality Indicators (PQIs), which consist of 13 sets of preventable hospitalization conditions. PQI hospitalization decreased from 154,565 to 151,168 per million hospitalizations during the study period (relative decrease, 2.2%; *P* = 0.041). Age-adjusted hospitalization rate increased for diabetes short-term complications (relative increase, 46.9%; *P* < 0.001) and lower-extremity amputations (relative increase, 15.1%; *P* = 0.035). Age stratified trends showed that hospitalization rates decreased significantly in all age-groups for diabetes short-term complications. For lower-extremity amputations, hospitalization rates increased significantly in younger age groups and decreased significantly in the older age groups. All other PQIs showed either decreasing or no change in trends. Adults aged 18–64 years should be the focus for future prevention attempts for diabetes complications. Identifying and acting on the factors responsible for these changes could help in reversing the concerning trends observed in this study. Existing strategies should focus on improving access to diabetes care and self-management.

## Introduction

During the past decade many reforms were proposed and implemented for improving primary care in the US. For example, delivery-system reforms such as the patient-centered medical home (PCMH) and the accountable care organization (ACO) initiatives have strengthened primary care through incentive and accountability-based services^1^. Primary care services have also expanded due to initiatives such as non-physician care coordinators, medical home services, and the Chronic Care Model interventions^2^. These have led to improved healthcare outcomes and eventually minimized avoidable downstream resource utilization and cost.

In 2000, the Agency for Healthcare Research and Quality (AHRQ) developed and published the Prevention Quality Indicators (PQIs), which consist of 13 sets of preventable hospitalization conditions, based on the International Classification of Diseases, 9th Revision, Clinical Modification (ICD-9-CM) diagnosis codes^3,4^. The PQIs have been validated by many studies that measured primary care performance, both nationally^5–7^ and locally^8–10^ within the US. The PQIs are conditions for which good outpatient care could significantly decrease the need for hospitalization, either for severe forms of the disease or its complications, and hence could be considered as a good proxy for effective primary care. Given the fact that obesity rates have significantly increased during the past two decades, this could have significantly affected the rates of many preventable hospitalization conditions. In the current study we aim to systematically assess national trends in preventable admissions that may inform policy makers as a proxy of primary care services.

## Methods

### Data source

The current study used the National Inpatient Sample (NIS) data collected during the period 2007–2016. AHRQ developed the NIS, which is the largest publicly available database for all-payer inpatient services in the US. The NIS contains discharge data from more than 1200 hospitals located in 45 states, collected and made available yearly since 1988. The NIS collects stratified sample of approximately 20% of US community hospital discharge data and is helpful in calculating national estimates. Individual hospitalizations are recorded in the NIS with one primary and many secondary diagnoses, after appropriate confidentiality and de-identification procedures, to ensure privacy of patients, physicians and hospitals. Individual hospitalization records also include other information such as demographics, insurance information, clinical procedures, comorbidities, length of stay, disposition status and total cost. Yearly quality assurance procedures are conducted to ensure the internal validity of the database.

### Study design and cohort

The current study is a retrospective analysis of NIS data. All hospitalizations for PQI conditions between 2007 and 2016 were identified using validated ICD-9-CM and ICD-10-CM codes and include acute and chronic PQI conditions^11^. Acute conditions include: dehydration; community acquired pneumonia; and urinary tract infection (UTI). Chronic conditions include: diabetes short-term complications; diabetes long-term complications; uncontrolled diabetes; lower-extremity amputation among patients with diabetes; hypertension; heart failure; asthma in younger adults 18–39 years; asthma and chronic obstructive pulmonary disease (COPD) in older adults ≥ 40 years; and perforated appendix. We did not include the PQI condition, low birth weight. All patients ≥ 18 years of age were included in the analyses. In addition, all hospitalizations with same admission and discharge dates were excluded from the analyses because these events do not truly represent a hospital stay^12^. Primary outcome of interest was hospitalization rates for PQI conditions during the study period. Secondary outcomes were length of stay, mortality, and hospitalization cost during the same period.

### Statistical analysis

Statistical analysis was performed using SAS 9.4 (SAS Institute, Cary, NC), which accounted for the complex survey design and clustering. We followed the guidelines developed by Khera and Krumholz for using NIS data^13^. The NIS was redesigned in 2012 to improve national estimates. To account for these changes, we used modified discharge weights for the years 2007–2011^14^. To estimate the significance of trends across the years, survey-weighted linear and logistic regression models were used for continuous and categorical variables, respectively. Annual percentage change (APC) for PQI hospitalization rate was calculated using Joinpoint Statistical Software (version 4.2.0.2), which uses permutation measures to find points of inflection. Linear and logistic regression were used to assess the P for trend. Joinpoint regression was used to find the inflection points and their significance. Yearly hospitalization rates for all individual PQI conditions were calculated by dividing the total number of PQI hospitalizations for these conditions by the estimated number of adults aged ≥ 18 years, obtained from US Census Bureau^15^. Subsequently, hospitalization rates for all PQI conditions were adjusted for age using the 2000 US standard population. We calculated both age-adjusted and age stratified rates. For age stratified rates, we categorized patients into 18–44, 45–64, 65–75, and ≥ 75 years. For asthma in the young, we categorized patients into 18–24, 25–30, 31–35, and 36–39 years. For asthma and COPD in older patients, we categorized patients into 40–50, 51–60, 61–70, and ≥ 71 years. These age categorizations were based on clinical relevance. In addition, we also calculated the composite hospitalization rate for acute conditions, chronic conditions, and total PQIs. Individual inpatient stay cost was calculated by multiplying total hospital charge and cost-to-charge ratio. The costs for each year was adjusted according to 2016 inflation levels released by the US Consumer Price Index^16^.

## Results

### Demographic characteristics

A total of 46,698,988 primary discharge diagnoses were reported as PQI hospitalization during 2007–2016. In 2016, the mean age of patients was 66.4 years and females constituted 54.8% of all PQI hospitalization (Table 1). The majority of the patients were white (66.3%), followed by blacks (16.7%) and Hispanics (8.3%). Slightly less than two thirds of the patients (64.5%) were Medicare beneficiaries, followed by private insurance (16.1%) and Medicaid (12.8%) coverages. Geographical distribution of patients showed that majority were admitted to hospitals in the South (42.5%), followed by the Midwest (22.9%), the Northeast (18.8%) and the West (15.8%). The majority of the patients were admitted to large (47.3%) and urban teaching hospitals (55.1%).

**Table 1:** Trends in demographic, hospital and clinical characteristics, 2007–2016.

	2007	2016	Relative change (%)	*P*_trend_
Unweighted sample	989,155	882,916		
Weighted sample	4,690,808	4,414,582		
Age, mean (SE)	67.3 (0.17)	66.4 (0.07)	− 1.3	< 0.001
Female, % (SE)	56.8% (0.16)	54.8% (0.09)	− 3.5	< 0.001
**Race, % (SE)**				< 0.001
White	53.7% (1.6)	66.3% (0.57)	23.5	
Black	9.6% (0.63)	16.7% (0.41)	74.0	
Hispanic	6.2% (0.58)	8.3% (0.32)	33.9	
Asian or Pacific Islander	1.1% (0.13)	1.7% (0.09)	54.5	
Native American	0.31% (0.05)	0.58% (0.05)	87.1	
Other	1.6% (0.20)	2.2% (0.13)	37.5	
Missing	27.6% (1.9)	4.1% (0.33)	− 85.1	
**Insurance type, % (SE)**				< 0.001
Medicare	65.9% (0.48)	64.5% (0.23)	− 2.1	
Medicaid	9.3% (0.29)	12.8% (0.19)	37.6	
Private insurance	18.0% (0.34)	16.1% (0.17)	− 10.6	
Self-pay	4.4% (0.27)	4.10% (0.10)	− 6.8	
No charge	0.49% (0.12)	0.44% (0.03)	− 10.2	
Other	1.8% (0.12)	1.9% (0.06)	5.6	
Missing	0.08% (0.01)	0.17% (0.03)	112.5	
**Median household income for patient’s zip code, % (SE)**				
1st Quartile	31.0% (1.10)	33.2% (0.56)	7.1	0.144
2nd Quartile	26.1% (0.82)	27.9% (0.40)	6.9	
3rd Quartile	22.4% (0.68)	20.8% (0.34)	− 7.1	
4th Quartile	18.2% (1.02)	16.0% (0.46)	− 12.1	
Missing	2.4% (0.21)	2.2% (0.10)	− 8.3	
**Hospital region, % (SE)**				0.984
Northeast	19.2% (1.7)	18.8% (0.73)	− 2.1	
Midwest	23.4% (1.6)	22.9% (0.76)	− 2.1	
South	41.7% (2.1)	42.5% (0.94)	1.9	
West	15.7% (1.4)	15.8% (0.60)	0.64	
**Hospital bed size, % (SE)**				< 0.001
Small	14.7% (1.0)	22.0% (0.61)	49.7	
Medium	25.7% (1.6)	30.7% (0.84)	19.5	
Large	59.5% (1.9)	47.3% (0.97)	− 20.5	
**Hospital status, % (SE)**				< 0.001
Rural	19.7% (1.2)	14.7% (0.51)	− 25.4	
Urban non-teaching	48.9% (2.1)	30.3% (0.83)	− 38.1	
Urban teaching	31.3% (2.2)	55.1% (0.91)	76.0	
Median cost of stay (in USD)	5817	7967	37.0	< 0.001
Total cost (in billion USD)	108.8	184.1	69.2	< 0.001

*SE* standard error.

### Trends in PQI hospitalization

PQI hospitalization decreased from 154,565 to 151,168 per million hospitalizations during the study period (relative decrease, 2.2%; *P* = 0.041). Age-adjusted hospitalization rates showed significantly decreasing trends for diabetes long-term complications (relative decrease, 12.5%; *P* < 0.001), uncontrolled diabetes (relative decrease, 37.0%; *P* = 0.002), hypertension (relative decrease, 20.3%; *P* = 0.010), heart failure (relative decrease, 22.7%; *P* < 0.001), asthma in younger adults (relative decrease, 28.7%; *P* < 0.001), pneumonia (relative decrease, 38.6%; *P* < 0.001), and perforated appendix (relative decrease, 21.8%; *P* < 0.001). Age stratified trends showed that hospitalization rates decreased significantly in all age-groups for diabetes long-term complications, uncontrolled diabetes, hypertension, heart failure, asthma in younger adults, pneumonia, and perforated appendix.

Age-adjusted hospitalization rates showed significantly increasing trends for diabetes short-term complications (relative increase, 46.9%; *P* < 0.001) and lower-extremity amputations (relative increase, 15.1%; *P* = 0.035). Age stratified trends showed that hospitalization rates increased significantly in all age-groups for diabetes short-term complications, whereas, for lower-extremity amputations, hospitalization rates increased significantly in the age groups 18–44 years and 45–64 years and decreased significantly in the age groups 65–74 years and ≥ 75 years.

**Figure 1:**
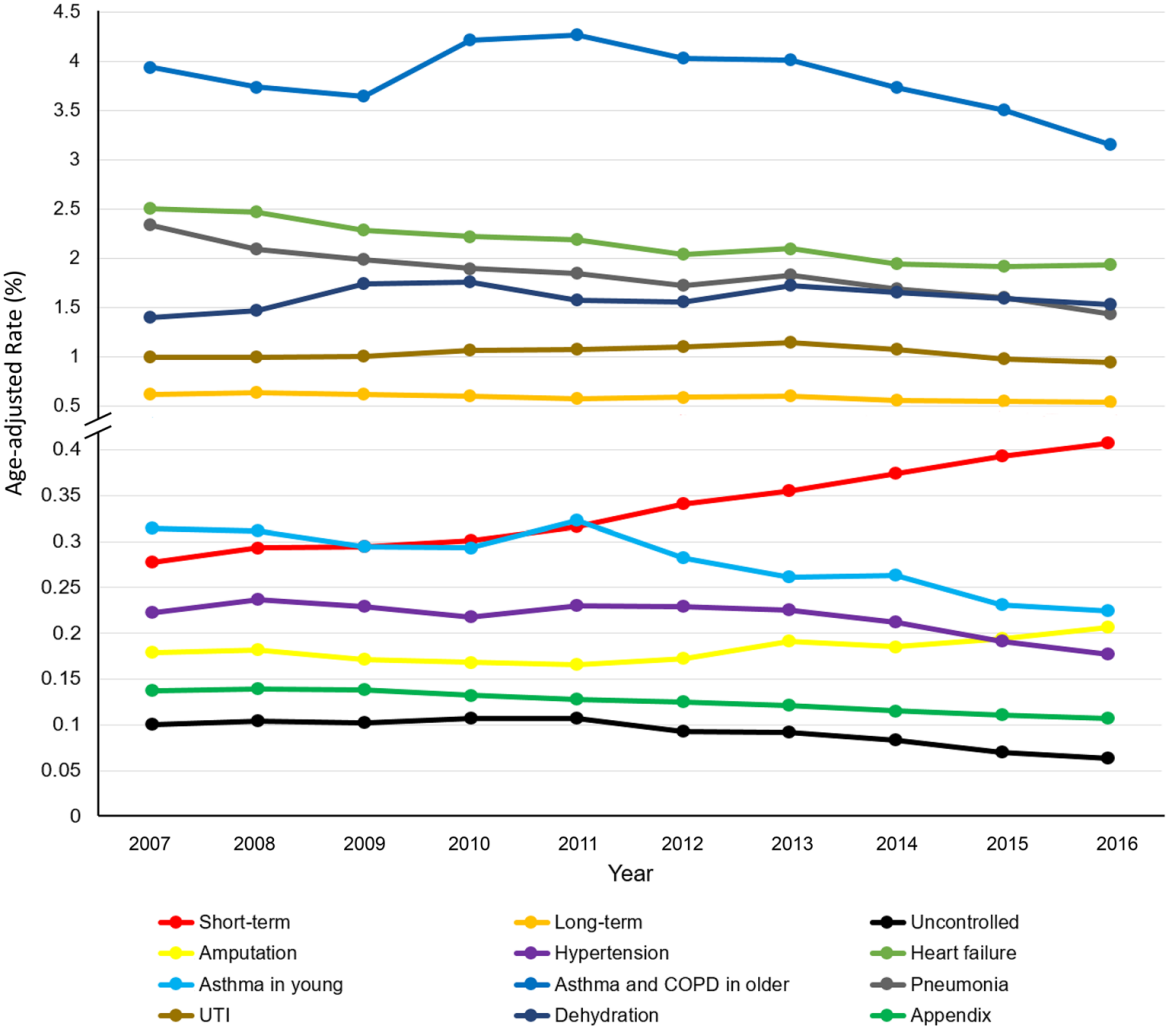
Trends of individual prevention quality indicator (PQI) hospitalizations in the United States, 2007–2016.

Age-adjusted hospitalization rates for asthma and COPD in older adults and UTI showed decreasing trends, whereas, for dehydration showed increasing trends, though they were not significant. Hospitalization rates for asthma and COPD in older adults increased significantly in the age groups 51–60 years, whereas, decreased significantly in the age groups 40–50 years, 61–70 years, and ≥ 71 years; for UTI increased significantly in the age groups ≥ 75 years, whereas, decreased significantly in the age groups 18–44 years, and 65–74 years; and for dehydration, decreased significantly in the age groups 18–44 years, whereas, increased significantly in the age groups 45–64 years, 65–74 years, and ≥ 75 years. Figure 1 shows trends for individual PQI conditions.

**Figure 2:**
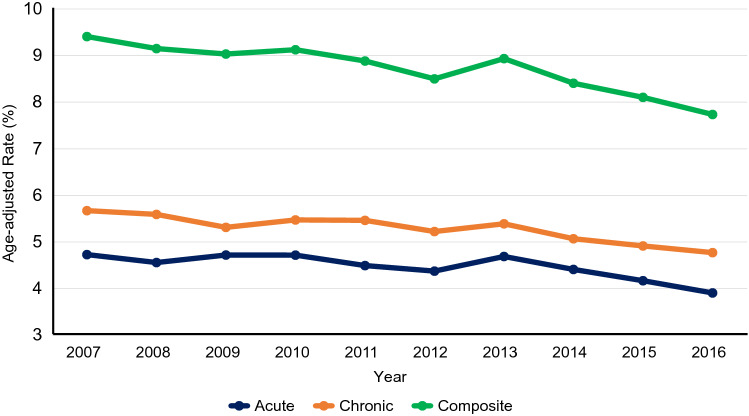
Trends of acute, chronic, and composite prevention quality indicator (PQI) hospitalizations in the United States, 2007–2016.

Age-adjusted hospitalization rates decreased significantly for acute conditions (relative decrease, 17.3%; *P* = 0.005), chronic conditions (relative decrease, 15.9%; *P* < 0.001), and overall conditions (relative decrease, 17.8%; *P* < 0.001). Age stratified trends showed that hospitalization rates decreased significantly in all age-groups for acute and overall conditions. Hospitalization rates due to chronic conditions increased significantly in the age group 18–44 years; whereas, decreased significantly in the age groups 45–64 years, 65–74 years, and ≥ 75 years. Figure 2 shows trends for acute, chronic, and composite conditions.

### Average annual percentage change

Hospitalization rate for many PQI conditions showed inflections in the trends. These inflections were located between the years 2007–2011. Trends after inflection increased significantly for diabetes short-term complications and lower-extremity amputation, while it decreased significantly for uncontrolled diabetes, hypertension, perforated appendix, and urinary tract infection. Table 2 shows average annual percent change and inflection points for PQI conditions using the joinpoint regression.

**Table 2:** Results of joinpoint trend analysis for PQI hospitalization rates in the United States, 2007–2016.

	Average annual percent change (95% CI)^b^	Trend 1^a^		Trend 2^a^
		End of trend 1	Annual percent change (95% CI)	Annual percent change (95% CI)
Diabetes short-term complications	4.6 (3.7, 5.5)	2010	2.5 (− 0.5, 5.6)	5.7 (4.8, 6.6)
Diabetes long-term complications	− 1.4 (− 2.1, − 0.7)	–	–	–
Uncontrolled diabetes	− 4.4 (− 6.6, − 2.3)	2011	1.7 (− 3.0, 6.6)	− 9.1 (− 12.5, − 5.6)
Lower-extremity amputation among diabetes	1.7 (− 0.1, 3.6)	2011	− 2.0 (− 6.0, 2.1)	4.8 (2.0, 7.7)
Hypertension	− 2.4 (− 4.1, − 0.7)	2013	0.2 (− 1.7, 2.0)	− 7.4 (− 12.4, − 2.0)
Heart failure	− 3.1 (− 3.7, − 2.5)	–	–	–
Asthma in younger adults 18–39 years	− 3.3 (− 4.7, − 1.9)	–	–	–
Asthma and COPD in older adults ≥ 40 years	− 1.8 (− 4.7, 1.2)	2013	1.1 (− 2.2, 4.4)	− 7.3 (− 16.1, 2.4)
Perforated appendix	− 2.4 (− 3.0, − 1.8)	2009	− 0.3 (− 3.5, 3.0)	− 3.0 (− 3.4, − 2.5)
Dehydration	1.5 (− 2.2, 5.4)	2009	11.9 (− 8.8, 37.2)	− 1.3 (− 3.7, 1.2)
Community acquired pneumonia	− 4.3 (− 5.3, − 3.3)	–	–	–
Urinary tract infection	− 0.5 (− 1.4, 0.5)	2013	2.5 (1.4, 3.6)	− 6.2 (− 9.1, − 3.2)
Acute conditions	− 1.6 (− 2.6, − 0.6)	–	–	–
Chronic conditions	− 1.7 (− 2.3, − 1.1)	–	–	–
All PQIs	− 1.8 (− 2.5, − 1.2)	–	–	–

*COPD* chronic obstructive pulmonary disease.

^a^The year 2007 always constitutes the starting point for Trend 1 if a significant joinpoint is present. The year 2016 always constitutes the ending point for Trend 2 if a significant joinpoint is present. The ending point of Trend 1 constitutes the starting point for Trend 2.

^b^The average annual percentage change is equal to the annual percentage change over the entire period if a joinpoint is not present.

### Length of stay, mortality and cost

During the study period median length of stay decreased from 3.3 to 3.0 days (*P* < 0.001) and mortality rate decreased from 2.7 to 1.8% (*P* < 0.001). Median cost of stay increased from $5817 to $7967 (*P* < 0.001) and total cost increased from $108.8 billion to $184.1 billion (*P* < 0.001) (Table 1).

## Discussion

During 2007–2016, hospitalizations rate decreased for acute conditions, chronic conditions, and overall PQIs, indicating generalized improvements in primary care services throughout the US. These findings are laudable given the fact that healthcare spending allocated to primary care in the US has been significantly lower compared to many other developed nations^17^. However, hospitalization rates increased for chronic conditions such as diabetes short-term complications and lower-extremity amputation, especially in younger population, which is concerning.

A previous trend study done by Wang et al.^7^ using NIS data during 1998–2006 reported that age-adjusted hospitalization rate for diabetes short-term complication decreased significantly. This finding was attributed to improvements in diabetes related primary care, aggressive treatment strategies, increased number of medications and treatment options, improved compliance to treatment regimes, and overall improvements in glycemic control over the years. The findings in our study paradoxically show increased age adjusted hospitalization rates for diabetes short-term complications, though treatment protocols and management strategies have significantly improved over the years. This increase observed in our study was contributed by substantial number of hospitalizations in the age group 18–44 years which increased during the years 2007–2016. The increasing rates of obesity and associated comorbidities could have contributed the increased number of hospitalizations in this age group. A study using National Health and Nutrition Examination Survey (NHANES) and the Behavioral Risk Factor Surveillance System (BRFSS) showed that participants in the age group 18–44 years had relatively poor glycemic control and were less likely than older participants to adhere to diabetes treatment and prevention protocols^18^. Similarly, another study which used the Nationwide Emergency Department Sample (NEDS) data during 2006–2011 reported that overall hyperglycemic crisis rates (which included ketoacidosis and hyperglycemic hyperosmolar states) increased significantly in the age group 18–44 years^19^. Such findings show that primary care services should focus on the age group 18–44 years. Early identification and treatment of microvascular and macrovascular complications should be achieved through advanced community health care services^20^. This could significantly decrease hospitalization rate and cost associated with treating complications. In addition, effective prevention strategies for decreasing obesity rates through dietary and lifestyle modification should be emphasized to decrease these hospitalizations.

Many studies have shown that lower-extremely amputation rate due to diabetes have decreased over the years^21^. Paradoxically, our analyses showed that hospitalization rate for lower-extremely amputation due to diabetes increased significantly during the study period. This paradoxical trend is driven by significant increase in amputation rate in the age group 18–44 and 45–64 years, though the reasons are not clear. Hence, we looked for hospitalizations rate due to diabetic foot ulcer, which is the most important risk factor for lower-extremity amputation^2,22^. The incidence of diabetic foot ulcer increased in the age groups 18–44 and 45–64 years (data not shown), which support the increased rate of lower-extremity amputations in these age groups. Similarly, the incidence of diabetic foot ulcers decreased in the age groups 65–74 and ≥ 75 years, which is also corresponding with the decreased rates of lower-extremity amputations in these age groups. In a study done by Li et al., using the National Hospital Discharge Survey and National Health Interview Survey, it was observed that hospitalization rate due to diabetes related lower-extremity amputation decreased in the age groups 64–75 years and ≥ 75 years, during 1988–2008, which is similar to our findings^23^. However, the same study also reported that hospitalization rate due to diabetes related lower-extremity amputation decreased in the age groups 40–64 years, which is contrary to our findings^23^. Increased lower-extremity amputation in young patients is concerning, considering the improvements in quality of care for diabetes over the years. Further studies are required to determine the exact cause for these findings (Supplementary Table 1).

Irrespective of our findings, our study has some limitations. Though we considered all hospitalizations as preventable, some of them may be non-preventable. Preventable and non-preventable hospitalizations could be differentiated only through case-by-case assessments. However, such case-by-case data are not available in the NIS, limiting our study findings. Though administrative databases are meticulously quality controlled, they are still susceptible to coding errors. ICD-9-CM and ICD-10-CM codes were used to identify the PQIs and there could be some coding errors leading to misclassification bias. The NIS deletes all personal identifiers to ensure confidentiality of the collected data. Patients who were readmitted were considered as independent new admission, obliterating the difference between index case and readmitted case. This could have led to overestimation of hospitalization rate. The NIS does not consider PQI hospitalization in federal hospitals such as Department of Defense, Department of Veterans Affairs, and Indian Health Service. Though these hospitals represent only a small fraction of the health care network, their non-inclusion decreases the external validity of our findings^24^.

During 2007–2016, hospitalization rate decreased for all PQI conditions, except diabetes short-term complications and lower-extremity amputation. Hospital length of stay and in-hospital mortality decreased, whereas, hospitalization cost increased during the same period. Increase in diabetes short-term complications was driven by the significant increase in the age group 18–44 years. Similarly, increase in lower-extremity amputation was driven by significant increase in amputation rate in the age group 18–44 and 45–64 years. These findings show that adults aged 18–64 years should be the focus for future prevention attempts for these complications. Identifying and acting on the factors responsible for these changes could help in reversing the concerning trends observed in this study. In the interim period, existing strategies should focus on improving access to diabetes care, increasing education for diabetes self-management, and preventive diabetes foot care for younger adults.

## Supplementary Information

Supplementary Tables.
