# Supplementary material for: Trends in quality of primary care in the United States, 2007–2016
Source: Sci Rep. 2022 Feb 7;12:1982. doi: 10.1038/s41598-022-06077-y (PMC8821600; doi:10.1038/s41598-022-06077-y)
Supplement: Supplementary file 1 — Supplementary Tables. [file 41598_2022_6077_MOESM1_ESM.docx]

Table 1. Diabetes short-term

|  | **2007** | **2008** | **2009** | **2010** | **2011** | **2012** | **2013** | **2014** | **2015** | **2016** | **Relative change** | ***P*_trend_** |
| --- | --- | --- | --- | --- | --- | --- | --- | --- | --- | --- | --- | --- |
| Total number of diabetes short-term complications-related preventable hospitalizations | 122197 | 130327 | 132013 | 136635 | 145069 | 158109 | 166034 | 176150 | 187295 | 195295 |  |  |
| Total number people >18 years in the US | 221992930 | 224622198 | 227211802 | 229989364 | 232637362 | 235153929 | 237804373 | 240400252 | 242887324 | 245273438 |  |  |
| Overall hospitalization rate, % | 0.055  (0.0016) | 0.058  (0.0016) | 0.058  (0.0016) | 0.059  (0.0016) | 0.062  (0.0016) | 0.067  (0.0017) | 0.070  (0.0017) | 0.073  (0.0017) | 0.077  (0.0018) | 0.080  (0.0018) | 45.4% | <.0001 |
| Age-adjusted hospitalization rate, % | 0.277  (0.0079) | 0.293  (0.0081) | 0.294  (0.0081) | 0.301  (0.0082) | 0.316  (0.0083) | 0.341  (0.0086) | 0.355  (0.0088) | 0.374  (0.009) | 0.393  (0.0091) | 0.407  (0.0093) | 46.9% | <.0001 |
| Hospitalization rate by age group, % |  |  |  |  |  |  |  |  |  |  |  |  |
| 18-44 years | 0.173  (0.0064) | 0.186  (0.0067) | 0.190  (0.0067) | 0.193  (0.0068) | 0.199  (0.0069) | 0.215  (0.0071) | 0.223  (0.0072) | 0.236  (0.0074) | 0.245  (0.0075) | 0.252  (0.0076) | 45.6% | <.0001 |
| 45-64 years | 0.073  (0.0039) | 0.077  (0.0039) | 0.077  (0.0039) | 0.080  (0.0039) | 0.086  (0.004) | 0.094  (0.0041) | 0.097  (0.0042) | 0.102  (0.0043) | 0.109(  0.0044) | 0.114  (0.0045) | 56.1% | <.0001 |
| 65-74 years | 0.016  (0.0019) | 0.016  (0.0019) | 0.015  (0.0018) | 0.015  (0.0018) | 0.017  (0.0019) | 0.018  (0.0019) | 0.019  (0.0019) | 0.019  (0.0019) | 0.021  (0.0019) | 0.023  (0.0019) | 43.7% | <.0001 |
| ≥75 years | 0.015  (0.0018) | 0.013  (0.0017) | 0.013  (0.0017) | 0.013  (0.0017) | 0.014  (0.0017) | 0.015  (0.0018) | 0.016  (0.0018) | 0.016  (0.0018) | 0.017  (0.0019) | 0.017  (0.0019) | 13.3% | <.0001 |
| Hospitalization rate by sex, % |  |  |  |  |  |  |  |  |  |  |  |  |
| Male | 0.135  (0.0055) | 0.143  (0.0056) | 0.140  (0.0055) | 0.145  (0.0056) | 0.150  (0.0057) | 0.162  (0.0059) | 0.170  (0.006) | 0.182  (0.0062) | 0.191  (0.0063) | 0.196  (0.0063) | 45.1% | <.0001 |
| Female | 0.140  (0.0056) | 0.147  (0.0057) | 0.150  (0.0057) | 0.151  (0.0057) | 0.162  (0.0059) | 0.174  (0.0061) | 0.179  (0.0061) | 0.184  (0.0062) | 0.195  (0.0063) | 0.202  (0.0064) | 44.2% | <.0001 |

Table 2. Diabetes long-term

|  | **2007** | **2008** | **2009** | **2010** | **2011** | **2012** | **2013** | **2014** | **2015** | **2016** | **Relative change** | ***P*_trend_** |
| --- | --- | --- | --- | --- | --- | --- | --- | --- | --- | --- | --- | --- |
| Total number of diabetes long-term complications-related preventable hospitalizations | 279208 | 293735 | 290590 | 288095 | 279256 | 290178 | 301519 | 284200 | 282550 | 284065 |  |  |
| Total number people >18 years in the US | 221992930 | 224622198 | 227211802 | 229989364 | 232637362 | 235153929 | 237804373 | 240400252 | 242887324 | 245273438 |  |  |
| Overall hospitalization rate, % | 0.126  (0.0024) | 0.131  (0.0024) | 0.128  (0.0024) | 0.125  (0.0023) | 0.120  (0.0023) | 0.123  (0.0023) | 0.127  (0.0023) | 0.118  (0.0022) | 0.116  (0.0022) | 0.116  (0.0022) | -7.9% | <.0001 |
| Age-adjusted hospitalization rate, % | 0.613  (0.0116) | 0.633  (0.0117) | 0.617  (0.0114) | 0.601  (0.0112) | 0.572  (0.0108) | 0.585  (0.0109) | 0.598  (0.0109) | 0.554  (0.0104) | 0.542  (0.0102) | 0.536  (0.0101) | -12.5% | <.0001 |
| Hospitalization rate by age group, % |  |  |  |  |  |  |  |  |  |  |  |  |
| 18-44 years | 0.103  (0.005) | 0.107  (0.005) | 0.112  (0.0052) | 0.109  (0.0051) | 0.112  (0.0051) | 0.12  (0.0053) | 0.121  (0.0053) | 0.115  (0.0052) | 0.113  (0.0051) | 0.113  (0.0051) | 9.7% | <.0001 |
| 45-64 years | 0.232  (0.0068) | 0.238  (0.0068) | 0.228  (0.0066) | 0.221  (0.0064) | 0.216  (0.0063) | 0.227  (0.0064) | 0.234  (0.0065) | 0.226  (0.0063) | 0.226  (0.0063) | 0.231  (0.0064) | -0.43% | <.0001 |
| 65-74 years | 0.130  (0.0054) | 0.133  (0.0055) | 0.128  (0.0053) | 0.122  (0.0051) | 0.112  (0.0048) | 0.109  (0.0046) | 0.112  (0.0046) | 0.10  (0.0042) | 0.096  (0.0041) | 0.093  (0.0039) | -28.5% | <.0001 |
| ≥75 years | 0.148  (0.0057) | 0.156  (0.0058) | 0.148  (0.0057) | 0.148  (0.0056) | 0.133  (0.0053) | 0.13  (0.0052) | 0.131  (0.0052) | 0.114  (0.0048) | 0.108  (0.0047) | 0.099  (0.0044) | -33.1% | <.0001 |
| Hospitalization rate by sex, % |  |  |  |  |  |  |  |  |  |  |  |  |
| Male | 0.303  (0.0082) | 0.313  (0.0083) | 0.303  (0.0082) | 0.299  (0.0081) | 0.279  (0.0077) | 0.284  (0.0078) | 0.287  (0.0078) | 0.263  (0.0074) | 0.249  (0.0072) | 0.244  (0.0071) | -19.5% | <.0001 |
| Female | 0.326  (0.0085) | 0.34  (0.0087) | 0.336  (0.0086) | 0.327  (0.0084) | 0.321  (0.0083) | 0.333  (0.0084) | 0.346  (0.0085) | 0.327  (0.0082) | 0.332  (0.0082) | 0.334  (0.0082) | 2.5% | 0.018 |

Table 3. Uncontrolled diabetes

|  | **2007** | **2008** | **2009** | **2010** | **2011** | **2012** | **2013** | **2014** | **2015** | **2016** | **Relative change** | ***P*_trend_** |
| --- | --- | --- | --- | --- | --- | --- | --- | --- | --- | --- | --- | --- |
| Total number of diabetes long-term complications-related preventable hospitalizations | 45139 | 48026 | 47931 | 50919 | 51696 | 45926 | 45717 | 42020 | 36150 | 32895 |  |  |
| Total number people >18 years in the US | 221992930 | 224622198 | 227211802 | 229989364 | 232637362 | 235153929 | 237804373 | 240400252 | 242887324 | 245273438 |  |  |
| Overall hospitalization rate, % | 0.02  (0.001) | 0.021  (0.001) | 0.021  (0.001) | 0.022  (0.001) | 0.022  (0.001) | 0.02  (0.0009) | 0.019  (0.0009) | 0.017  (0.0009) | 0.015  (0.0008) | 0.013  (0.0007) | -35.0% | <.0001 |
| Age-adjusted hospitalization rate, % | 0.10  (0.0047) | 0.104  (0.0048) | 0.102  (0.0047) | 0.107  (0.0048) | 0.107  (0.0047) | 0.093  (0.0044) | 0.092  (0.0043) | 0.083  (0.0041) | 0.07  (0.0037) | 0.063  (0.0035) | -37.0% | 0.002 |
| Hospitalization rate by age group, % |  |  |  |  |  |  |  |  |  |  |  |  |
| 18-44 years | 0.029  (0.0026) | 0.031  (0.0027) | 0.031  (0.0027) | 0.031  (0.0027) | 0.031  (0.0027) | 0.027  (0.0025) | 0.026  (0.0025) | 0.024  (0.0024) | 0.02  (0.0022) | 0.018  (0.0021) | -37.9% | <.0001 |
| 45-64 years | 0.039  (0.0028) | 0.04  (0.0028) | 0.039  (0.0028) | 0.042  (0.0028) | 0.042  (0.0028) | 0.037  (0.0026) | 0.036  (0.0026) | 0.033  (0.0024) | 0.029  (0.0023) | 0.025  (0.0021) | -35.9% | <.0001 |
| 65-74 years | 0.015  (0.0019) | 0.016  (0.0019) | 0.015  (0.0018) | 0.016  (0.0018) | 0.016  (0.0018) | 0.014  (0.0017) | 0.014  (0.0016) | 0.012  (0.0015) | 0.01  (0.0013) | 0.009  (0.0012) | -40.0% | <.0001 |
| ≥75 years | 0.017  (0.0019) | 0.017  (0.002) | 0.017  (0.0019) | 0.018  (0.002) | 0.018  (0.002) | 0.016  (0.0018) | 0.016  (0.0018) | 0.015  (0.0017) | 0.012  (0.0016) | 0.011  (0.0015) | -35.3% | <.0001 |
| Hospitalization rate by sex, % |  |  |  |  |  |  |  |  |  |  |  |  |
| Male | 0.052  (0.0034) | 0.054  (0.0035) | 0.054  (0.0034) | 0.058  (0.0036) | 0.056  (0.0035) | 0.049  (0.0032) | 0.048  (0.0032) | 0.044  (0.003) | 0.037  (0.0028) | 0.034  (0.0026) | -34.6% | <.0001 |
| Female | 0.05  (0.0033) | 0.053  (0.0034) | 0.052  (0.0034) | 0.053  (0.0034) | 0.055  (0.0034) | 0.048  (0.0032) | 0.048  (0.0032) | 0.043  (0.003) | 0.038  (0.0028) | 0.033  (0.0026) | -34.0% | <.0001 |

Table 4. Amputation-related

|  | **2007** | **2008** | **2009** | **2010** | **2011** | **2012** | **2013** | **2014** | **2015** | **2016** | **Relative change** | ***P*_trend_** |
| --- | --- | --- | --- | --- | --- | --- | --- | --- | --- | --- | --- | --- |
| Total number of diabetes long-term complications-related preventable hospitalizations | 82066 | 84965 | 81530 | 81527 | 82243 | 87076 | 98338 | 96930 | 103430 | 112005 |  |  |
| Total number people >18 years in the US | 221992930 | 224622198 | 227211802 | 229989364 | 232637362 | 235153929 | 237804373 | 240400252 | 242887324 | 245273438 |  |  |
| Overall hospitalization rate, % | 0.037  (0.0013) | 0.038  (0.0013) | 0.036  (0.0013) | 0.035  (0.0012) | 0.035  (0.0012) | 0.037  (0.0013) | 0.041  (0.0013) | 0.04  (0.0013) | 0.043  (0.0013) | 0.046  (0.0014) | 24.3% | <.0001 |
| Age-adjusted hospitalization rate, % | 0.179  (0.0062) | 0.182  (0.0062) | 0.171  (0.006) | 0.168  (0.0059) | 0.166  (0.0058) | 0.172  (0.0058) | 0.191  (0.0061) | 0.185  (0.0059) | 0.194  (0.006) | 0.206  (0.0062) | 15.1% | 0.035 |
| Hospitalization rate by age group, % |  |  |  |  |  |  |  |  |  |  |  |  |
| 18-44 years | 0.016  (0.0019) | 0.017  (0.002) | 0.016  (0.0019) | 0.016  (0.002) | 0.016  (0.0019) | 0.017  (0.002) | 0.021  (0.0022) | 0.02  (0.0022) | 0.022  (0.0023) | 0.022  (0.0023) | 37.5% | <.0001 |
| 45-64 years | 0.072  (0.0038) | 0.075  (0.0039) | 0.07  (0.0037) | 0.069  (0.0036) | 0.071  (0.0036) | 0.077  (0.0037) | 0.085  (0.0039) | 0.085  (0.0039) | 0.092  (0.004) | 0.1  (0.0042) | 38.9% | <.0001 |
| 65-74 years | 0.045  (0.0032) | 0.044  (0.0032) | 0.042  (0.0031) | 0.042  (0.003) | 0.039  (0.0028) | 0.04  (0.0028) | 0.043  (0.0029) | 0.041  (0.0027) | 0.042  (0.0027) | 0.044  (0.0027) | -2.2% | <.0001 |
| ≥75 years | 0.046  (0.0032) | 0.045  (0.0032) | 0.043  (0.003) | 0.041  (0.003) | 0.039  (0.0029) | 0.037  (0.0028) | 0.042  (0.003) | 0.038  (0.0028) | 0.038  (0.0028) | 0.039  (0.0028) | -15.2% | <.0001 |
| Hospitalization rate by sex, % |  |  |  |  |  |  |  |  |  |  |  |  |
| Male | 0.067  (0.0039) | 0.068  (0.0039) | 0.065  (0.0038) | 0.062  (0.0037) | 0.059  (0.0036) | 0.059  (0.0036) | 0.067  (0.0038) | 0.064  (0.0036) | 0.066  (0.0037) | 0.068  (0.0037) | 1.5% | 0.0862 |
| Female | 0.118  (0.0051) | 0.121  (0.0052) | 0.115  (0.005) | 0.115  (0.005) | 0.117  (0.005) | 0.125  (0.0051) | 0.14  (0.0054) | 0.137  (0.0053) | 0.147  (0.0055) | 0.159  (0.0057) | 34.7% | <.0001 |

Table 5. Hypertension

|  | **2007** | **2008** | **2009** | **2010** | **2011** | **2012** | **2013** | **2014** | **2015** | **2016** | **Relative change** | ***P*_trend_** |
| --- | --- | --- | --- | --- | --- | --- | --- | --- | --- | --- | --- | --- |
| Total number of diabetes long-term complications-related preventable hospitalizations | 101175 | 109994 | 107889 | 104296 | 111900 | 113289 | 112748 | 108395 | 99050 | 93035 |  |  |
| Total number people >18 years in the US | 221992930 | 224622198 | 227211802 | 229989364 | 232637362 | 235153929 | 237804373 | 240400252 | 242887324 | 245273438 |  |  |
| Overall hospitalization rate, % | 0.046  (0.0014) | 0.049  (0.0015) | 0.047  (0.0014) | 0.045  (0.0014) | 0.048  (0.0014) | 0.048  (0.0014) | 0.047  (0.0014) | 0.045  (0.0014) | 0.041  (0.0013) | 0.038  (0.0012) | -17.4% | <.0001 |
| Age-adjusted hospitalization rate, % | 0.222  (0.007) | 0.237  (0.0072) | 0.229  (0.007) | 0.218  (0.0068) | 0.23  (0.0069) | 0.229  (0.0068) | 0.225  (0.0067) | 0.212  (0.0065) | 0.191  (0.0061) | 0.177  (0.0058) | -20.3% | 0.010 |
| Hospitalization rate by age group, % |  |  |  |  |  |  |  |  |  |  |  |  |
| 18-44 years | 0.041  (0.0031) | 0.044  (0.0033) | 0.044  (0.0033) | 0.04  (0.0031) | 0.044  (0.0032) | 0.044  (0.0032) | 0.042  (0.0031) | 0.038  (0.003) | 0.036  (0.0029) | 0.035  (0.0028) | -14.6% | <.0001 |
| 45-64 years | 0.08  (0.004) | 0.089  (0.0042) | 0.085  (0.004) | 0.08  (0.0039) | 0.086  (0.004) | 0.088  (0.004) | 0.082  (0.0038) | 0.08  (0.0038) | 0.074  (0.0036) | 0.068  (0.0035) | -15.0% | <.0001 |
| 65-74 years | 0.04  (0.003) | 0.041  (0.003) | 0.038  (0.0029) | 0.037  (0.0028) | 0.038  (0.0028) | 0.036  (0.0027) | 0.037  (0.0027) | 0.034  (0.0025) | 0.028  (0.0022) | 0.027  (0.0021) | -32.5% | <.0001 |
| ≥75 years | 0.061  (0.0037) | 0.063  (0.0037) | 0.062  (0.0037) | 0.06  (0.0036) | 0.062  (0.0036) | 0.061  (0.0036) | 0.064  (0.0037) | 0.06  (0.0035) | 0.053  (0.0033) | 0.048  (0.0031) | -21.3% | <.0001 |
| Hospitalization rate by sex, % |  |  |  |  |  |  |  |  |  |  |  |  |
| Male | 0.143  (0.0057) | 0.154  (0.0059) | 0.15  (0.0058) | 0.144  (0.0056) | 0.15  (0.0057) | 0.15  (0.0056) | 0.15  (0.0056) | 0.141  (0.0054) | 0.125  (0.0051) | 0.116  (0.0049) | -18.9% | <.0001 |
| Female | 0.085  (0.0044) | 0.091  (0.0045) | 0.087  (0.0044) | 0.082  (0.0042) | 0.091  (0.0044) | 0.091  (0.0044) | 0.087  (0.0043) | 0.085  (0.0042) | 0.079  (0.004) | 0.074  (0.0039) | -12.9% | <.0001 |

Table 6. Heart failure

|  | **2007** | **2008** | **2009** | **2010** | **2011** | **2012** | **2013** | **2014** | **2015** | **2016** | **Relative change** | ***P*_trend_** |
| --- | --- | --- | --- | --- | --- | --- | --- | --- | --- | --- | --- | --- |
| Total number of diabetes long-term complications-related preventable hospitalizations | 1127715 | 1133080 | 1061956 | 1049890 | 1051655 | 997111 | 1003405 | 951145 | 960079 | 989060 |  |  |
| Total number people >18 years in the US | 221992930 | 224622198 | 227211802 | 229989364 | 232637362 | 235153929 | 237804373 | 240400252 | 242887324 | 245273438 |  |  |
| Overall hospitalization rate, % | 0.508  (0.0048) | 0.504  (0.0047) | 0.467  (0.0045) | 0.456  (0.0044) | 0.452  (0.0044) | 0.424  (0.0042) | 0.431  (0.0043) | 0.405  (0.0041) | 0.404  (0.0041) | 0.413  (0.0041) | -18.7% | <.0001 |
| Age-adjusted hospitalization rate, % | 2.501  (0.0222) | 2.472  (0.022) | 2.281  (0.0211) | 2.218  (0.0206) | 2.187  (0.0203) | 2.04  (0.0195) | 2.095  (0.0199) | 1.943  (0.0191) | 1.918  (0.0188) | 1.933  (0.0187) | -22.7% | <.0001 |
| Hospitalization rate by age group, % |  |  |  |  |  |  |  |  |  |  |  |  |
| 18-44 years | 0.11  (0.0051) | 0.117  (0.0053) | 0.112  (0.0052) | 0.103  (0.0049) | 0.102  (0.0049) | 0.101  (0.0049) | 0.097  (0.0049) | 0.095  (0.0048) | 0.096  (0.0048) | 0.101  (0.0049) | -8.2% | <.0001 |
| 45-64 years | 0.496  (0.01) | 0.506  (0.0099) | 0.474  (0.0095) | 0.446  (0.0091) | 0.45  (0.0091) | 0.43  (0.0088) | 0.421  (0.0087) | 0.402  (0.0085) | 0.418  (0.0086) | 0.436  (0.0088) | -12.1% | <.0001 |
| 65-74 years | 0.548  (0.0109) | 0.53  (0.0107) | 0.483  (0.0101) | 0.452  (0.0096) | 0.446  (0.0093) | 0.404  (0.0088) | 0.403  (0.0087) | 0.368  (0.0081) | 0.361  (0.0078) | 0.36  (0.0076) | -34.3% | <.0001 |
| ≥75 years | 1.346  (0.0158) | 1.319  (0.0156) | 1.211  (0.015) | 1.218  (0.015) | 1.189  (0.0148) | 1.106  (0.0143) | 1.173  (0.0149) | 1.077(0.0143) | 1.044  (0.014) | 1.036  (0.0138) | -23.0% | <.0001 |
| Hospitalization rate by sex, % |  |  |  |  |  |  |  |  |  |  |  |  |
| Male | 1.328  (0.0171) | 1.305  (0.0169) | 1.204  (0.0161) | 1.171  (0.0158) | 1.145  (0.0155) | 1.063  (0.0149) | 1.083  (0.0151) | 1.005  (0.0145) | 0.989  (0.0143) | 0.999  (0.0143) | -24.8% | <.0001 |
| Female | 1.212  (0.0163) | 1.217  (0.0162) | 1.132  (0.0156) | 1.112  (0.0153) | 1.115  (0.0153) | 1.057  (0.0148) | 1.074  (0.0151) | 1.018  (0.0146) | 1.033  (0.0146) | 1.063  (0.0147) | -12.3% | <.0001 |

Table 7. Asthma in young

|  | **2007** | **2008** | **2009** | **2010** | **2011** | **2012** | **2013** | **2014** | **2015** | **2016** | **Relative change** | ***P*_trend_** |
| --- | --- | --- | --- | --- | --- | --- | --- | --- | --- | --- | --- | --- |
| Total number of diabetes long-term complications-related preventable hospitalizations | 55228 | 55002 | 52112 | 52251 | 57864 | 50467 | 46975 | 47765 | 42290 | 41410 |  |  |
| Total number people >18 years in the US | 89498846 | 89939251 | 90485379 | 91219210 | 91761484 | 92037533 | 92530415 | 93246062 | 94082854 | 94902312 |  |  |
| Overall hospitalization rate, % | 0.062  (0.0026) | 0.061  (0.0026) | 0.058  (0.0025) | 0.057  (0.0025) | 0.063  (0.0026) | 0.055(0.0024) | 0.051(0.0023) | 0.051(0.0023) | 0.045  (0.0022) | 0.044  (0.0021) | -29.0% | <.0001 |
| Age-adjusted hospitalization rate, % | 0.314  (0.0134) | 0.311  (0.0133) | 0.294  (0.0129) | 0.293  (0.0128) | 0.323  (0.0134) | 0.282  (0.0126) | 0.261  (0.0121) | 0.263  (0.0121) | 0.231  (0.0113) | 0.224  (0.0111) | -28.7% | <.0001 |
| Hospitalization rate by age group, % |  |  |  |  |  |  |  |  |  |  |  |  |
| 18-24 years | 0.064  (0.0057) | 0.066  (0.0058) | 0.058  (0.0055) | 0.06  (0.0055) | 0.068  (0.0058) | 0.058  (0.0054) | 0.055  (0.0052) | 0.058  (0.0053) | 0.049  (0.0049) | 0.046  (0.0047) | -28.1% | <.0001 |
| 25-30 years | 0.072  (0.0064) | 0.069  (0.0061) | 0.066  (0.0059) | 0.065  (0.0059) | 0.074  (0.0062) | 0.064  (0.0057) | 0.062  (0.0056) | 0.06  (0.0055) | 0.054  (0.0052) | 0.053  (0.0051) | -26.4% | <.0001 |
| 31-35 years | 0.086  (0.007) | 0.082  (0.0069) | 0.079  (0.0069) | 0.081  (0.007) | 0.086  (0.0072) | 0.074  (0.0066) | 0.07  (0.0063) | 0.069  (0.0062) | 0.064  (0.0059) | 0.06  (0.0057) | -30.2% | <.0001 |
| 36-39 years | 0.092  (0.0075) | 0.094  (0.0075) | 0.09  (0.0074) | 0.086  (0.0072) | 0.095  (0.0076) | 0.086  (0.0073) | 0.074  (0.0069) | 0.077  (0.007) | 0.064  (0.0064) | 0.066  (0.0065) | -28.3% | <.0001 |
| Hospitalization rate by sex, % |  |  |  |  |  |  |  |  |  |  |  |  |
| Male | 0.215  (0.0109) | 0.209  (0.0108) | 0.194  (0.0103) | 0.196  (0.0103) | 0.215  (0.0108) | 0.187  (0.01) | 0.172  (0.0096) | 0.172  (0.0096) | 0.152  (0.009) | 0.147  (0.0088) | -31.6% | <.0001 |
| Female | 0.093  (0.0072) | 0.096  (0.0073) | 0.093  (0.0072) | 0.089  (0.007) | 0.1  (0.0074) | 0.087  (0.0069) | 0.081  (0.0066) | 0.084  (0.0067) | 0.073  (0.0062) | 0.072  (0.0061) | -22.6% | <.0001 |

Table 8. Asthma and COPD in older

|  | **2007** | **2008** | **2009** | **2010** | **2011** | **2012** | **2013** | **2014** | **2015** | **2016** | **Relative change** | ***P*_trend_** |
| --- | --- | --- | --- | --- | --- | --- | --- | --- | --- | --- | --- | --- |
| Total number of diabetes long-term complications-related preventable hospitalizations | 798734 | 763057 | 753363 | 884502 | 909184 | 875641 | 893791 | 855655 | 825299 | 764465 |  |  |
| Total number people >18 years in the US | 132494084 | 134682947 | 136726423 | 138770154 | 140875878 | 143116396 | 145273958 | 147154190 | 148804470 | 150371126 |  |  |
| Overall hospitalization rate, % | 0.603  (0.0067) | 0.567  (0.0065) | 0.551  (0.0063) | 0.637  (0.0068) | 0.645  (0.0067) | 0.612  (0.0065) | 0.615  (0.0065) | 0.581  (0.0063) | 0.555  (0.0061) | 0.508  (0.0058) | -15.8% | <.0001 |
| Age-adjusted hospitalization rate, % | 3.939  (0.0458) | 3.737  (0.0449) | 3.645  (0.0445) | 4.218  (0.0473) | 4.268  (0.0479) | 4.034  (0.0464) | 4.011  (0.0455) | 3.73  (0.0438) | 3.504  (0.0422) | 3.154  (0.0399) | -19.9% | 0.149 |
| Hospitalization rate by age group, % |  |  |  |  |  |  |  |  |  |  |  |  |
| 40-50 years | 0.843  (0.0265) | 0.864  (0.0271) | 0.88  (0.0276) | 0.946  (0.0289) | 1.044  (0.0306) | 0.977  (0.0297) | 0.887  (0.0283) | 0.855  (0.0277) | 0.781  (0.0267) | 0.713  (0.0257) | -15.4% | <.0001 |
| 51-60 years | 0.247  (0.0064) | 0.237  (0.0062) | 0.239  (0.0062) | 0.265  (0.0064) | 0.287  (0.0066) | 0.278  (0.0065) | 0.276  (0.0064) | 0.277  (0.0064) | 0.269  (0.0063) | 0.262  (0.0062) | 6.1% | <.0001 |
| 61-70 years | 0.88  (0.0191) | 0.824  (0.0184) | 0.786  (0.0178) | 0.899  (0.0186) | 0.914  (0.0184) | 0.853  (0.0175) | 0.861  (0.0174) | 0.775  (0.016) | 0.716  (0.015) | 0.638  (0.0139) | -27.5% | <.0001 |
| ≥71 years | 1.969  (0.0314) | 1.812  (0.03) | 1.742  (0.0294) | 2.107  (0.0319) | 2.022  (0.0312) | 1.926  (0.0304) | 1.986  (0.0305) | 1.824  (0.0292) | 1.737  (0.0283) | 1.541  (0.0266) | -21.7% | <.0001 |
| Hospitalization rate by sex, % |  |  |  |  |  |  |  |  |  |  |  |  |
| Male | 1.849  (0.0261) | 1.736  (0.0251) | 1.681  (0.0245) | 1.935  (0.0261) | 1.971  (0.0261) | 1.873  (0.0253) | 1.898  (0.0253) | 1.774  (0.0243) | 1.686  (0.0236) | 1.545  (0.0225) | -16.4% | <.0001 |
| Female | 1.167  (0.0206) | 1.1  (0.0199) | 1.078  (0.0195) | 1.257  (0.0209) | 1.261  (0.0207) | 1.192  (0.02) | 1.185  (0.0198) | 1.139  (0.0193) | 1.092  (0.0188) | 1.002  (0.0179) | -14.1% | <.0001 |

Table 9. Pneumonia

|  | **2007** | **2008** | **2009** | **2010** | **2011** | **2012** | **2013** | **2014** | **2015** | **2016** | **Relative change** | ***P*_trend_** |
| --- | --- | --- | --- | --- | --- | --- | --- | --- | --- | --- | --- | --- |
| Total number of diabetes long-term complications-related preventable hospitalizations | 1052509 | 957291 | 921479 | 893914 | 885360 | 837994 | 873051 | 825850 | 798089 | 730800 |  |  |
| Total number people >18 years in the US | 221992930 | 224622198 | 227211802 | 229989364 | 232637362 | 235153929 | 237804373 | 240400252 | 242887324 | 245273438 |  |  |
| Overall hospitalization rate, % | 0.474  (0.0046) | 0.426  (0.0043) | 0.406  (0.0042) | 0.389  (0.0041) | 0.381  (0.004) | 0.356  (0.0039) | 0.375  (0.004) | 0.351  (0.0039) | 0.336  (0.0038) | 0.305  (0.0036) | -35.7% | <.0001 |
| Age-adjusted hospitalization rate, % | 2.338  (0.0218) | 2.093  (0.0206) | 1.983  (0.02) | 1.893  (0.0194) | 1.847  (0.0191) | 1.72  (0.0183) | 1.824  (0.019) | 1.69  (0.0181) | 1.601  (0.0175) | 1.436  (0.0165) | -38.6% | <.0001 |
| Hospitalization rate by age group, % |  |  |  |  |  |  |  |  |  |  |  |  |
| 18-44 years | 0.239  (0.0075) | 0.219  (0.0072) | 0.225  (0.0073) | 0.218  (0.0072) | 0.246  (0.0076) | 0.208  (0.007) | 0.21  (0.0071) | 0.196  (0.0069) | 0.175  (0.0065) | 0.164  (0.0062) | -31.4% | <.0001 |
| 45-64 years | 0.494  (0.0099) | 0.46  (0.0095) | 0.45  (0.0093) | 0.426  (0.0089) | 0.439  (0.009) | 0.404  (0.0085) | 0.416  (0.0086) | 0.396  (0.0084) | 0.376  (0.0082) | 0.355  (0.0079) | -28.1% | <.0001 |
| 65-74 years | 0.467  (0.0101) | 0.421  (0.0096) | 0.392  (0.0091) | 0.357  (0.0086) | 0.348  (0.0083) | 0.321  (0.0079) | 0.333  (0.0079) | 0.305  (0.0074) | 0.288  (0.007) | 0.253  (0.0064) | -45.8% | <.0001 |
| ≥75 years | 1.137  (0.0148) | 0.993  (0.0139) | 0.916  (0.0133) | 0.892  (0.0131) | 0.814  (0.0126) | 0.787  (0.0123) | 0.865  (0.0131) | 0.792  (0.0125) | 0.761  (0.0122) | 0.664  (0.0113) | -41.6% | <.0001 |
| Hospitalization rate by sex, % |  |  |  |  |  |  |  |  |  |  |  |  |
| Male | 1.281  (0.0168) | 1.144  (0.0158) | 1.086  (0.0153) | 1.042  (0.0149) | 1.019  (0.0147) | 0.952  (0.0141) | 1.000  (0.0145) | 0.933  (0.0139) | 0.892  (0.0136) | 0.808  (0.0129) | -36.9% | <.0001 |
| Female | 1.088  (0.0155) | 0.987  (0.0146) | 0.942  (0.0142) | 0.900  (0.0138) | 0.883  (0.0136) | 0.830  (0.0131) | 0.876  (0.0136) | 0.823  (0.0131) | 0.788  (0.0128) | 0.716  (0.0121) | -34.2% | <.0001 |

Table 10. UTI

|  | **2007** | **2008** | **2009** | **2010** | **2011** | **2012** | **2013** | **2014** | **2015** | **2016** | **Relative change** | ***P*_trend_** |
| --- | --- | --- | --- | --- | --- | --- | --- | --- | --- | --- | --- | --- |
| Total number of diabetes long-term complications-related preventable hospitalizations | 442447 | 451577 | 457727 | 494959 | 504905 | 524353 | 532811 | 510860 | 475399 | 466345 |  |  |
| Total number people >18 years in the US | 221992930 | 224622198 | 227211802 | 229989364 | 232637362 | 235153929 | 237804373 | 240400252 | 242887324 | 245273438 |  |  |
| Overall hospitalization rate, % | 0.199  (0.003) | 0.201  (0.003) | 0.201  (0.003) | 0.215  (0.0031) | 0.217  (0.0031) | 0.223  (0.0031) | 0.229  (0.0031) | 0.217  (0.003) | 0.2  (0.0029) | 0.195  (0.0028) | -2.0% | <.0001 |
| Age-adjusted hospitalization rate, % | 0.99  (0.0146) | 0.997  (0.0146) | 0.998  (0.0145) | 1.064  (0.0148) | 1.072  (0.0148) | 1.098  (0.0149) | 1.14  (0.0153) | 1.072  (0.0147) | 0.979  (0.014) | 0.942  (0.0136) | -4.8% | 0.9618 |
| Hospitalization rate by age group, % |  |  |  |  |  |  |  |  |  |  |  |  |
| 18-44 years | 0.171  (0.0064) | 0.171  (0.0064) | 0.17  (0.0063) | 0.174  (0.0064) | 0.176  (0.0065) | 0.181  (0.0065) | 0.173  (0.0065) | 0.165  (0.0063) | 0.151  (0.006) | 0.145  (0.0059) | -15.2% | <.0001 |
| 45-64 years | 0.159  (0.0057) | 0.16  (0.0056) | 0.159  (0.0055) | 0.163  (0.0055) | 0.168  (0.0056) | 0.173  (0.0056) | 0.175  (0.0056) | 0.168  (0.0055) | 0.156  (0.0053) | 0.154  (0.0052) | -3.1% | 0.8276 |
| 65-74 years | 0.151  (0.0059) | 0.151  (0.0058) | 0.155  (0.0058) | 0.16  (0.0058) | 0.161  (0.0057) | 0.164  (0.0057) | 0.163  (0.0056) | 0.151  (0.0052) | 0.136  (0.0049) | 0.132  (0.0047) | -12.6% | <.0001 |
| ≥75 years | 0.509  (0.0103) | 0.516  (0.0103) | 0.515  (0.0103) | 0.568  (0.0107) | 0.567  (0.0107) | 0.58  (0.0107) | 0.629  (0.0114) | 0.588  (0.0109) | 0.536  (0.0104) | 0.511  (0.0101) | 0.39% | <.0001 |
| Hospitalization rate by sex, % |  |  |  |  |  |  |  |  |  |  |  |  |
| Male | 0.717  (0.0126) | 0.727  (0.0127) | 0.727  (0.0126) | 0.787  (0.013) | 0.79  (0.013) | 0.809  (0.013) | 0.833  (0.0132) | 0.792  (0.0129) | 0.731  (0.0123) | 0.713  (0.0121) | -0.56% | <.0001 |
| Female | 0.28  (0.0079) | 0.279  (0.0078) | 0.281  (0.0078) | 0.29  (0.0079) | 0.296  (0.0079) | 0.308  (0.008) | 0.31  (0.0082) | 0.293  (0.0079) | 0.268  (0.0075) | 0.259  (0.0073) | -7.5% | <.0001 |

Table 11. Dehydration

|  | **2007** | **2008** | **2009** | **2010** | **2011** | **2012** | **2013** | **2014** | **2015** | **2016** | **Relative change** | ***P*_trend_** |
| --- | --- | --- | --- | --- | --- | --- | --- | --- | --- | --- | --- | --- |
| Total number of diabetes long-term complications-related preventable hospitalizations | 628,925 | 669,623 | 805695 | 828916 | 753697 | 756390 | 824524 | 804065 | 791144 | 776035 |  |  |
| Total number people >18 years in the US | 221,992,930 | 224,622,198 | 227,211,802 | 229,989,364 | 232,637,362 | 235,153,929 | 237,804,373 | 240,400,252 | 242,887,324 | 245,273,438 |  |  |
| Overall hospitalization rate, % | 0.283  (0.0036) | 0.298  (0.0036) | 0.355  (0.0039) | 0.36  (0.004) | 0.324  (0.0037) | 0.322  (0.0037) | 0.354  (0.0039) | 0.342  (0.0038) | 0.333  (0.0037) | 0.324  (0.0037) | 14.5% | <.0001 |
| Age-adjusted hospitalization rate, % | 1.4  (0.0173) | 1.468  (0.0176) | 1.738  (0.0189) | 1.759  (0.0189) | 1.575  (0.0178) | 1.557  (0.0176) | 1.723  (0.0185) | 1.648  (0.018) | 1.589  (0.0175) | 1.528  (0.0171) | 9.1% | 0.5441 |
| Hospitalization rate by age group, % |  |  |  |  |  |  |  |  |  |  |  |  |
| 18-44 years | 0.248  (0.0077) | 0.251  (0.0077) | 0.256  (0.0078) | 0.267  (0.0079) | 0.249  (0.0077) | 0.248  (0.0076) | 0.241  (0.0076) | 0.242  (0.0076) | 0.23  (0.0074) | 0.226  (0.0073) | -8.9% | <.0001 |
| 45-64 years | 0.316  (0.008) | 0.339  (0.0081) | 0.397  (0.0087) | 0.408  (0.0087) | 0.377  (0.0083) | 0.38  (0.0083) | 0.408  (0.0085) | 0.403  (0.0085) | 0.396  (0.0084) | 0.391  (0.0083) | 23.7% | <.0001 |
| 65-74 years | 0.245  (0.0074) | 0.261  (0.0076) | 0.323  (0.0083) | 0.322  (0.0081) | 0.287  (0.0076) | 0.276  (0.0073) | 0.309  (0.0077) | 0.287  (0.0072) | 0.282  (0.0069) | 0.271  (0.0066) | 10.6% | <.0001 |
| ≥75 years | 0.591  (0.0111) | 0.616  (0.0112) | 0.762  (0.0123) | 0.763  (0.0123) | 0.662  (0.0115) | 0.654  (0.0113) | 0.765  (0.0124) | 0.716  (0.012) | 0.681  (0.0116) | 0.64  (0.0112) | 8.3% | <.0001 |
| Hospitalization rate by sex, % |  |  |  |  |  |  |  |  |  |  |  |  |
| Male | 0.852  (0.0138) | 0.889  (0.014) | 1.026  (0.0149) | 1.043  (0.0149) | 0.934  (0.0141) | 0.914  (0.0138) | 0.995  (0.0145) | 0.955  (0.0141) | 0.919  (0.0138) | 0.885  (0.0135) | 3.9% | 0.0022 |
| Female | 0.564  (0.0112) | 0.602  (0.0115) | 0.747  (0.0127) | 0.759  (0.0127) | 0.686  (0.012) | 0.695  (0.012) | 0.776  (0.0128) | 0.755  (0.0126) | 0.746  (0.0125) | 0.733  (0.0123) | 30.0% | <.0001 |

Table 12. Appendix

|  | **2007** | **2008** | **2009** | **2010** | **2011** | **2012** | **2013** | **2014** | **2015** | **2016** | **Relative change** | ***P*_trend_** |
| --- | --- | --- | --- | --- | --- | --- | --- | --- | --- | --- | --- | --- |
| Total number of diabetes long-term complications-related preventable hospitalizations | 61463 | 62931 | 63182 | 61483 | 60412 | 59764 | 58461 | 56455 | 55005 | 53735 |  |  |
| Total number people >18 years in the US | 221,992,930 | 224,622,198 | 227,211,802 | 229,989,364 | 232,637,362 | 235,153,929 | 237,804,373 | 240,400,252 | 242,887,324 | 245,273,438 |  |  |
| Overall hospitalization rate, % | 0.028  (0.0011) | 0.028  (0.0011) | 0.028  (0.0011) | 0.027  (0.0011) | 0.026  (0.0011) | 0.025  (0.001) | 0.025  (0.001) | 0.023  (0.001) | 0.023  (0.001) | 0.022  (0.0009) | -21.4% | <.0001 |
| Age-adjusted hospitalization rate, % | 0.137  (0.0055) | 0.139  (0.0056) | 0.138  (0.0055) | 0.132  (0.0054) | 0.128  (0.0052) | 0.125  (0.0051) | 0.121  (0.005) | 0.115  (0.0049) | 0.111  (0.0048) | 0.107  (0.0047) | -21.8% | <.0001 |
| Hospitalization rate by age group, % |  |  |  |  |  |  |  |  |  |  |  |  |
| 18-44 years | 0.064  (0.0039) | 0.066  (0.004) | 0.065  (0.0039) | 0.062  (0.0038) | 0.059  (0.0037) | 0.057  (0.0037) | 0.055  (0.0036) | 0.053  (0.0035) | 0.05  (0.0034) | 0.048  (0.0033) | -25.0% | <.0001 |
| 45-64 years | 0.047  (0.0031) | 0.047  (0.003) | 0.046  (0.003) | 0.044  (0.0029) | 0.043  (0.0028) | 0.043  (0.0028) | 0.042  (0.0027) | 0.04  (0.0027) | 0.039  (0.0026) | 0.038  (0.0026) | -19.1% | <.0001 |
| 65-74 years | 0.014  (0.0018) | 0.015  (0.0018) | 0.015  (0.0018) | 0.016  (0.0018) | 0.015  (0.0017) | 0.014  (0.0017) | 0.014  (0.0016) | 0.013  (0.0015) | 0.012  (0.0015) | 0.012  (0.0014) | -14.2% | <.0001 |
| ≥75 years | 0.012  (0.0016) | 0.012  (0.0016) | 0.012  (0.0016) | 0.011  (0.0015) | 0.011  (0.0015) | 0.012  (0.0016) | 0.011  (0.0015) | 0.01  (0.0015) | 0.01  (0.0014) | 0.009  (0.0014) | -25.0% | <.0001 |
| Hospitalization rate by sex, % |  |  |  |  |  |  |  |  |  |  |  |  |
| Male | 0.06  (0.0037) | 0.06  (0.0037) | 0.059  (0.0036) | 0.058  (0.0036) | 0.056  (0.0035) | 0.055  (0.0034) | 0.054  (0.0034) | 0.051  (0.0033) | 0.05  (0.0032) | 0.048  (0.0031) | -20.0% | <.0001 |
| Female | 0.077  (0.0042) | 0.079  (0.0042) | 0.079  (0.0041) | 0.074  (0.004) | 0.073  (0.004) | 0.071  (0.0039) | 0.068  (0.0038) | 0.066  (0.0037) | 0.063  (0.0036) | 0.061  (0.0035) | -20.7% | <.0001 |

Table 13. Acute

|  | **2007** | **2008** | **2009** | **2010** | **2011** | **2012** | **2013** | **2014** | **2015** | **2016** | **Relative change** | ***P*_trend_** |
| --- | --- | --- | --- | --- | --- | --- | --- | --- | --- | --- | --- | --- |
| Total number of diabetes long-term complications-related preventable hospitalizations | 2123882 | 2078491 | 2184901 | 2217789 | 2143962 | 2118737 | 2230386 | 2140776 | 2064633 | 1973181 |  |  |
| Total number people >18 years in the US | 221992930 | 224622198 | 227211802 | 229989364 | 232637362 | 235153929 | 237804373 | 240400252 | 242887324 | 245273438 |  |  |
| Overall hospitalization rate, % | 0.957  (0.0065) | 0.925  (0.0064) | 0.962  (0.0065) | 0.964  (0.0064) | 0.922  (0.0063) | 0.901  (0.0062) | 0.959  (0.0064) | 0.911  (0.0062) | 0.869  (0.006) | 0.823  (0.0058) | -14.0% | <.0001 |
| Age-adjusted hospitalization rate, % | 4.728  (0.0298) | 4.558  (0.0292) | 4.72  (0.0294) | 4.717  (0.0291) | 4.494  (0.0285) | 4.375  (0.0279) | 4.687  (0.0288) | 4.41  (0.0279) | 4.169  (0.027) | 3.906  (0.0261) | -17.4% | 0.0053 |
| Hospitalization rate by age group, % |  |  |  |  |  |  |  |  |  |  |  |  |
| 18-44 years | 0.658  (0.0125) | 0.642  (0.0123) | 0.651  (0.0124) | 0.658  (0.0124) | 0.671  (0.0125) | 0.638  (0.0122) | 0.624  (0.0122) | 0.603  (0.012) | 0.556  (0.0115) | 0.534  (0.0112) | -18.8% | <.0001 |
| 45-64 years | 0.969  (0.0138) | 0.958  (0.0135) | 1.006  (0.0137) | 0.998  (0.0135) | 0.984  (0.0133) | 0.956  (0.013) | 0.999  (0.0132) | 0.968  (0.013) | 0.929  (0.0128) | 0.9  (0.0125) | -7.1% | <.0001 |
| 65-74 years | 0.863  (0.0134) | 0.833  (0.0131) | 0.87  (0.0132) | 0.839  (0.0127) | 0.796  (0.0122) | 0.761  (0.0118) | 0.804  (0.012) | 0.743  (0.0112) | 0.706  (0.0107) | 0.657  (0.0101) | -23.9% | <.0001 |
| ≥75 years | 2.237  (0.019) | 2.125  (0.0186) | 2.193  (0.0187) | 2.223  (0.0187) | 2.042  (0.0181) | 2.02  (0.018) | 2.26  (0.019) | 2.096  (0.0184) | 1.978  (0.0179) | 1.814  (0.0172) | -18.9% | <.0001 |
| Hospitalization rate by sex, % |  |  |  |  |  |  |  |  |  |  |  |  |
| Male | 2.851  (0.0247) | 2.76  (0.0242) | 2.839  (0.0243) | 2.873  (0.0243) | 2.744  (0.0237) | 2.675  (0.0233) | 2.828  (0.0239) | 2.68  (0.0232) | 2.542  (0.0225) | 2.405  (0.0218) | -15.6% | <.0001 |
| Female | 1.932  (0.0204) | 1.868  (0.02) | 1.969  (0.0204) | 1.95  (0.0201) | 1.865  (0.0196) | 1.832  (0.0193) | 1.962  (0.0202) | 1.87  (0.0196) | 1.803  (0.0191) | 1.708  (0.0186) | -11.6% | <.0001 |

Table 14. Chronic

|  | **2007** | **2008** | **2009** | **2010** | **2011** | **2012** | **2013** | **2014** | **2015** | **2016** | **Relative change** | ***P*_trend_** |
| --- | --- | --- | --- | --- | --- | --- | --- | --- | --- | --- | --- | --- |
| Total number of diabetes long-term complications-related preventable hospitalizations | 2563932 | 2568888 | 2481666 | 2601417 | 2642778 | 2568466 | 2612361 | 2507221 | 2477383 | 2449821 |  |  |
| Total number people >18 years in the US | 221992930 | 224622198 | 227211802 | 229989364 | 232637362 | 235153929 | 237804373 | 240400252 | 242887324 | 245273438 |  |  |
| Overall hospitalization rate, % | 1.155  (0.0072) | 1.144  (0.0071) | 1.092  (0.0069) | 1.131  (0.007) | 1.136  (0.0069) | 1.092  (0.0068) | 1.123  (0.0069) | 1.066  (0.0067) | 1.043  (0.0066) | 1.022  (0.0065) | -11.5% | <.0001 |
| Age-adjusted hospitalization rate, % | 5.672  (0.0326) | 5.591  (0.0322) | 5.314  (0.0314) | 5.473  (0.0314) | 5.464  (0.0312) | 5.224  (0.0305) | 5.389  (0.031) | 5.069  (0.03) | 4.915  (0.0294) | 4.772  (0.0289) | -15.9% | <.0001 |
| Hospitalization rate by age group, % |  |  |  |  |  |  |  |  |  |  |  |  |
| 18-44 years | 0.679  (0.0126) | 0.707  (0.0129) | 0.701  (0.0128) | 0.686  (0.0127) | 0.715  (0.0129) | 0.71  (0.0129) | 0.714  (0.0131) | 0.71  (0.013) | 0.694  (0.0128) | 0.694  (0.0128) | 2.2% | <.0001 |
| 45-64 years | 1.528  (0.0172) | 1.534  (0.017) | 1.479  (0.0165) | 1.502  (0.0164) | 1.557  (0.0165) | 1.527  (0.0162) | 1.525  (0.0162) | 1.487  (0.016) | 1.475  (0.0159) | 1.472  (0.0159) | -3.7% | <.0001 |
| 65-74 years | 1.256  (0.0158) | 1.21  (0.0154) | 1.133  (0.0148) | 1.159  (0.0147) | 1.146  (0.0143) | 1.061  (0.0137) | 1.078  (0.0136) | 0.977  (0.0127) | 0.932  (0.0121) | 0.883  (0.0115) | -29.7% | <.0001 |
| ≥75 years | 2.209  (0.0189) | 2.14  (0.0186) | 2.001  (0.0181) | 2.126  (0.0184) | 2.046  (0.0181) | 1.927  (0.0177) | 2.071  (0.0185) | 1.895  (0.0178) | 1.814  (0.0174) | 1.723  (0.0169) | -22.0% | <.0001 |
| Hospitalization rate by sex, % |  |  |  |  |  |  |  |  |  |  |  |  |
| Male | 3.176  (0.0259) | 3.12  (0.0256) | 2.965  (0.0248) | 3.084  (0.0252) | 3.078  (0.025) | 2.94  (0.0243) | 3.012  (0.0246) | 2.833  (0.0238) | 2.728  (0.0233) | 2.641  (0.0228) | -16.8% | <.0001 |
| Female | 2.598  (0.0235) | 2.598  (0.0234) | 2.495  (0.0228) | 2.571  (0.023) | 2.601  (0.0229) | 2.521  (0.0225) | 2.602  (0.0231) | 2.498  (0.0225) | 2.487  (0.0223) | 2.467  (0.0221) | -5.0% | <.0001 |

Table 15. Composite

|  | **2007** | **2008** | **2009** | **2010** | **2011** | **2012** | **2013** | **2014** | **2015** | **2016** | **Relative change** | ***P*_trend_** |
| --- | --- | --- | --- | --- | --- | --- | --- | --- | --- | --- | --- | --- |
| Total number of diabetes long-term complications-related preventable hospitalizations | 4245029 | 4195516 | 4208450 | 4323892 | 4281488 | 4162566 | 4309624 | 4136816 | 4066296 | 3956342 |  |  |
| Total number people >18 years in the US | 221992930 | 224622198 | 227211802 | 229989364 | 232637362 | 235153929 | 237804373 | 240400252 | 242887324 | 245273438 |  |  |
| Overall hospitalization rate, % | 1.912  (0.0092) | 1.868  (0.009) | 1.852  (0.0089) | 1.88  (0.009) | 1.84  (0.0088) | 1.77  (0.0086) | 1.853  (0.0088) | 1.759  (0.0086) | 1.712  (0.0084) | 1.65  (0.0082) | -13.7% | <.0001 |
| Age-adjusted hospitalization rate, % | 9.409  (0.0389) | 9.151  (0.0384) | 9.035  (0.038) | 9.125  (0.0377) | 8.885  (0.0375) | 8.501  (0.0366) | 8.935  (0.0372) | 8.406  (0.0363) | 8.103  (0.0356) | 7.736  (0.0349) | -17.8% | <.0001 |
| Hospitalization rate by age group, % |  |  |  |  |  |  |  |  |  |  |  |  |
| 18-44 years | 1.166  (0.0165) | 1.177  (0.0166) | 1.182  (0.0166) | 1.171  (0.0165) | 1.211  (0.0168) | 1.166  (0.0164) | 1.166  (0.0166) | 1.148  (0.0164) | 1.099  (0.0161) | 1.084  (0.0159) | -7.0% | <.0001 |
| 45-64 years | 2.337  (0.0209) | 2.332  (0.0206) | 2.325  (0.0203) | 2.33  6(0.0202) | 2.373  (0.0201) | 2.31  (0.0197) | 2.349  (0.0198) | 2.287  (0.0196) | 2.247  (0.0194) | 2.218  (0.0192) | -5.1% | <.0001 |
| 65-74 years | 1.968  (0.0188) | 1.892  (0.0184) | 1.848  (0.018) | 1.837  (0.0176) | 1.78  (0.0171) | 1.658  (0.0164) | 1.72  (0.0165) | 1.569  (0.0154) | 1.501  (0.0148) | 1.407  (0.0141) | -28.5% | <.0001 |
| ≥75 years | 3.937  (0.0211) | 3.75  (0.0209) | 3.679  (0.0208) | 3.781  (0.0208) | 3.522  (0.0206) | 3.367  (0.0204) | 3.701  (0.021) | 3.403  (0.0207) | 3.256  (0.0204) | 3.026  (0.0199) | -23.1% | <.0001 |
| Hospitalization rate by sex, % |  |  |  |  |  |  |  |  |  |  |  |  |
| Male | 5.309  (0.0328) | 5.152  (0.0322) | 5.076  (0.0318) | 5.169  (0.0319) | 5.032  (0.0313) | 4.806  (0.0305) | 5.006  (0.0311) | 4.721  (0.0302) | 4.539  (0.0295) | 4.334  (0.0287) | -18.4% | <.0001 |
| Female | 4.249  (0.0295) | 4.186  (0.0291) | 4.183  (0.0289) | 4.23  (0.0289) | 4.17  (0.0286) | 4.045  (0.028) | 4.254  (0.0289) | 4.075  (0.0282) | 4.021  (0.0279) | 3.915  (0.0274) | -7.9% | <.0001 |
